# Impacts of Rumen Degradable or Undegradable Protein Supplementation with or without Salt on Nutrient Digestion, and VFA Concentrations

**DOI:** 10.3390/ani11113011

**Published:** 2021-10-20

**Authors:** Marley Manoukian, Timothy DelCurto, Janessa Kluth, Tanner Carlisle, Noah Davis, Makae Nack, Samuel Wyffels, Abe Scheaffer, Megan Van Emon

**Affiliations:** 1 Department of Animal and Range Sciences, Montana State University, Bozeman, MT 59717, USA; marley.manoukian@montana.edu (M.M.); timothy.delcurto@montana.edu (T.D.); jakluth@outlook.com (J.K.); tcarlisle@deseretranches.com (T.C.); noahdavis3@montana.edu (N.D.); makaenack@gmail.com (M.N.); 2 Northern Agricultural Research Center, Montana State University, Havre, MT 59501, USA; samwyffels@montana.edu; 3 SweetPro LLC, Walhalla, ND 58282, USA; abe.scheaffer@sweetpro.com

**Keywords:** beef cattle, delivery method, digestion, rumen degradable protein, rumen undegradable protein

## Abstract

**Simple Summary:**

Ruminant animals have two specific protein requirements: the protein required by the animal, and the protein required by the microorganisms that exist within the rumen of the animal. These requirements are satisfied by rumen undegradable (RUP) and rumen degradable protein (RDP), respectively. Within the rumen, RDP is hydrolyzed, while RUP is digested and absorbed in the small intestine. As these proteins are digested differently, we studied their impact on the digestion process of low-quality forage. Overall, we found that a RDP supplement, when fed in a self-fed form, may enhance the digestion and use of low-quality forages. This may assist producers in selecting supplementation programs for their operation.

**Abstract:**

The objectives of this study were to evaluate the effect of differences in protein type and delivery method on rumen dynamics and nutrient digestion. Cows were allotted to rumen degradable protein (RDP) or rumen undegradable protein (RUP) and self-fed (SF) salt-limited pressed blocks or hand-fed (HF) loose supplement, resulting in four dietary treatments. There was a delivery effect (*p* = 0.04) on neutral detergent fiber (NDF) intake, as the SF animals consumed more NDF than HF animals. The RDP-SF animals had greater NDF digestibility (*p* = 0.04) and water intake (*p* = 0.03) than the three other treatments. Supplement intake displayed a protein type effect (*p* = 0.03), as RDP-supplemented animals consumed more supplement on a g·kg body weight (BW)^−1^ d^−1^ basis than RUP animals. There was an effect of protein type (*p* = 0.02) and delivery method (*p* = 0.03) on fluid flow rate, with RUP and HF cows having greater liquid flow rates. Ruminal pH was lower (*p* < 0.01) in RDP-HF cows than RDP-SF cows at all hours, except 4-h post-feeding. RDP-SF animals had the greatest (*p* < 0.01) concentrations of ruminal ammonia. Valerate ruminal concentrations were greater (*p* = 0.04) in RDP supplemented animals compared to RUP supplemented animals. In conclusion, self-fed supplements containing RDP may enhance the use of low-quality forages and increase ruminal ammonia concentrations.

## 1. Introduction

Beef cattle production is important in the western United States. The arid environment of the region causes forage to have seasonal deficiencies, and it can often be low in protein [1], which is why supplementing cattle consuming these low-quality forages with protein is important. The protein required by beef cattle can be separated into two specific requirements: the protein needs of the rumen microorganisms, and the needs of the individual animal [2]. Rumen degradable protein (RDP) provides the microorganisms in the rumen with a source of N and is required for the synthesis of microbial crude protein. Rumen undegradable protein (RUP) is not hydrolyzed upon entering the rumen and, as a result, may be digested and absorbed in the small intestine.

A positive relationship exists between RDP supplementation and forage utilization [3]. The N source that RDP provides to rumen microorganisms allows them to grow [4]. An increase in microbial growth leads to an increase in microbial activity, and therefore an increase in microbial N flow to the small intestine [5]. An increase in microbial activity may result in an increase in forage digestion. Unlike RDP, RUP does not provide rumen microbes with a N source. The differences in how RDP and RUP are digested may result in a difference in microbial populations among treatments. 

Since microbes utilize the protein available within the rumen to grow [4], an increase in microbial growth and activity increases the production of volatile fatty acids (VFA) within the rumen [3,5]. In a study by Wickersham et al. [4], beef steers were supplemented with increasing amounts of RDP. Overall VFA concentrations were increased with increasing amounts of RDP, specifically with acetate declining and propionate increasing. It is probable that we could see similar results with changes in VFA concentrations. Additionally, ammonia is a product of excess protein in the diet and is ultimately absorbed and released as the waste product urea [6]. Within the rumen, RDP is broken down into peptides and then amino acids [7]. It is likely that increases in protein available to the rumen will result in an increase of ammonia.

The impacts of RUP and RDP supplementation on fiber digestion vary. Digestibility of acid detergent fiber (ADF) was not impacted when first calf heifers were supplemented with RDP or RUP postpartum [8]. Similarly, Pina et al. [9] observed that the level of RUP supplementation did not impact dry matter intake (DMI) or digestibility of dry matter (DM), organic matter (OM), neutral detergent fiber crude protein (NDFcp), or total digestible nutrients (TDN) when Nellore heifers were fed RUP at either 25 or 40% of crude protein (CP) [9]. In contrast, non-supplemented periparturient cows, compared to cows supplemented with RUP, had lower NDF digestibility [10]. 

These differences in how each type, and amount, of protein is digested have the potential to cause differences in nutrient digestion, VFA concentrations, ammonia concentrations, and rumen microbiology profiles. Therefore, the objectives of this study were (1) to evaluate the effect of differences in protein type and delivery method on rumen dynamics, and (2) evaluate the effect of differences in protein type and delivery method on nutrient digestion. 

## 2. Materials and Methods

The experimental procedures described herein were approved by the Agriculture Animal Care and Use Committee of Montana State University (#2020-AA05). Eight two-year old (477.86 ± 35.28 kg) and eight three-year old (607.59 ± 48.11 kg) rumen fistulated cows were used in a 2 × 2 factorial design for a 22-day digestion study. Shrunk weights were collected on day 1. Cows were stratified by BW, within age and within stratum, and assigned to 1 of 16 pens (1 cow per pen), and one of four dietary treatments (Table 1): (1) self-fed, salt-limited pressed supplement block, containing rumen undegradable protein (RUP-SF; SweetPro^®^ Premium supplements, Walhalla, ND, USA); (2) hand-fed, loose supplement, containing RUP (RUP-HF; SweetPro^®^ Premium supplements, Walhalla, ND, USA); (3) self-fed, salt-limited pressed supplement block, containing rumen degradable protein (RDP-SF); and (4) hand-fed, loose supplement, containing RDP (RDP-HF). Self-fed supplements were formulated to be isonitrogenous, isocaloric, and contain 25% salt, as salt has been shown to be an effective intake limiter [11]. These supplements were also formulated as press blocks, as this form of supplement has also been utilized to control intake. The RDP supplements were formulated to be similar; however, differences in ingredients were required to form the RDP-SF blocks, which led to the differences in fiber content. Additionally, due to the RUP supplement being commercially available, salt was not removed from the RUP-HF supplement. The 22-day period included a 14-day adaptation period, 7-day total dry matter intake and total fecal collection, and 2-day collection of rumen fluid samples for ruminal and microbial profiles and rumen dry matter content. 

The experimental period was previously described by, and was modified from, Bohner, et al. [12]. Low-quality forage (7.2% CP; Table 2) was fed at 120% of the average previous 3-day intake. Supplement blocks were fed ad libitum and intake measured daily by weight disappearance. Loose supplements were hand-fed at a rate of 0.91 kg cow^−1^ d^−1^. Water was offered ab libitum to all 16 cows in stock tanks, and intake was measured by weight disappearance. Forage and supplement were provided at 08:00 each day. Indwelling wireless data transmission boluses (smaXtec animal care, GmbH, Graz, Austria) were placed in the reticulorumen and used to monitor pH and temperature (Table S1) at 10-min intervals during the 7-day collection period. 

During the 7-day collection period, orts were collected daily from day 16 to day 22 for determination of DMI. Total fecal output was measured for determination of DM digestibility. Forage, supplement, orts, fecal, and water samples were collected from day 15 to day 21. Forage, orts, and supplement samples were dried at 55 °C for 48 h, ground to pass a 1 mm screen (Wiley Mill Model 4, Thomas Scientific, Swedesboro, NJ, USA), and stored for further analysis. Fecal samples were dried at 55 °C for 96 h, ground to pass a 1 mm screen, and stored for further analysis. Forage, supplement, orts, and fecal samples were analyzed for NDF (AOAC, 2005) using the Ankom 200 Fiber Analyzer (Ankom Co., Fairport, NY, USA). Total tract dry matter digestibility and NDF digestibility (Table S2) was determined. 

On day 21, each cow was intraruminally pulse-dosed with 286.25 mg mL^−1^ of Cr-EDTA in a 250-mL aqueous solution [13], prior to feeding at hour 0. The Cr marker was administered throughout the rumen. Rumen fluid (approximately 115 mL) was collected using a suction strainer [14] (19-mm diameter, 1.6-mm mesh), immediately prior to dosing and at 4, 8, 12, 18, and 24-h post-dosing. Rumen fluid was stored at −20 °C for Cr, VFA, and ammonia analyses (Table S3). Rumen fluid samples were analyzed for ammonia concentrations using methods similar to those described by Sigma Technical Bulletin #640 [15], Chaney and Marbach [16], Horn and Squire [17], and Weichselbaum et al. [18]. Rumen fluid samples were analyzed for individual VFA concentrations using a gas chromatography procedure similar to that described by Baumgardt [19], Supleco Inc. Bulletin 749E [20], Byers [21], and Fritz and Schenk [22]. Chromium concentrations were analyzed with atomic absorption using an air/acetylene flame. Ruminal liquid volume and liquid dilution rates were estimated by regressing the natural logarithm of Cr concentrations against sampling time [23]. On day 20, an additional subsample of rumen fluid was collected immediately prior to dosing of Cr-EDTA. This rumen fluid was frozen immediately at −20 °C for rumen microbiota analysis. 

Additionally, on day 22, ruminal DM and undigestible NDF (uNDF) were determined by manually removing reticulorumen contents 5 h post-feeding. Total rumen contents were weighed, mixed by hand, and subsampled in duplicate. The remaining ruminal contents were immediately replaced in the cow. Rumen samples were weighed, dried in a forced-air oven at 55 °C for 96 h and reweighed for DM. Dried rumen samples were composited and ground to pass a 1 mm screen in a Wiley Mill (Model 4, Thomas Scientific, Swedesboro, NJ, USA). Samples were sent to a commercial laboratory (Dairy One, Ithaca, NY, USA) and analyzed for uNDF. Undigestible NDF (Table S2) values were utilized to determine passage rates. 

The effects of protein type and delivery method on daily intake, water consumption, and digesta kinetics were analyzed using analysis of variance (ANOVA) with a generalized linear model, for a 2 × 2 factorial design. The effects of protein type and delivery method on VFAs, pH, and ammonia were analyzed using ANOVA with generalized mixed models, for a repeated measure analysis. An individual cow was considered a random intercept for VFAs and ammonia, as there were repeated measurements for each individual. An individual cow nested within a day was considered a random intercept for pH, as ruminal pH was collected hourly for each individual over the course of the 7-day collection period. Data were plotted and log-transformed if needed, to satisfy assumptions of normality and homogeneity of variance. An alpha of ≤0.05 was considered significant, and tendencies were considered at alpha ≤0.1. Means were separated using the Tukey method, when *p* ≤ 0.05. All statistical analyses were performed in R [24].

## 3. Results

### 3.1. Intake and Digestibility

There were no protein type or delivery method effects (*p* ≥ 0.24; Table 3) on dry matter intake expressed in g/kg BW/d, or forage intake expressed as both kg^−1^ d^−1^ and g kg BW^−1^ d^−1^. There were no protein type or delivery method effects (*p* ≥ 0.12) on uNDF fill in kg or uNDF fill in g kg BW^−1^, nor uNDF retention or passage. There was a delivery method effect (*p* = 0.04) on NDF intake, as the SF animals consumed more NDF than the HF animals. There was a delivery × protein type interaction (*p* ≤ 0.04) for both NDF digestibility and water intake, where RDP-SF had greater NDF digestibility (*p* = 0.05) and water intake (*p* < 0.01) than RDP-HF, however there were no differences in NDF digestibility (*p* = 0.32) or water intake (*p* = 0.98) between RUP-SF and RUP-HF. As we would expect, due to our delivery system, animals on the SF method had increased (*p* = 0.01) supplement intake in kg d^−1^ compared to HF animals that were limit-fed supplement. Supplement intake in g·kg BW^−1^ d^−1^ displayed a protein type effect (*p* = 0.03), as RDP supplemented animals consumed more supplement on a g·kg BW^-1^ basis than RUP animals. Delivery method tended (*p* = 0.07) to effect dry matter intake in kg d^−1^, as SF consumed more compared to HF. Dry matter digestibility tended (*p* = 0.08) to display a delivery × protein type interaction, in which RDP-SF had greater DM digestibility compared to RDP-HF, while RUP-HF had improved DM digestibility compared to RUP-SF.

There were no protein type or delivery method effects (*p* ≥ 0.25; Table 4) on ruminal DM volume. There was an effect (*p* = 0.02) of protein type on fluid flow rate, as RUP supplemented animals had higher rates compared to RDP supplemented animals, 2.83 and 2.03 L h^−1^, respectively. There was also an effect (*p* = 0.03) of delivery on fluid flow rate; however, post-hoc means separation analysis showed no difference between delivery methods. Protein type tended (*p* = 0.07) to have an effect on ruminal fluid volume, as RUP-supplemented cows tended to have increased volume compared to RDP animals. 

### 3.2. Rumen Dynamics

**Figure 1:**
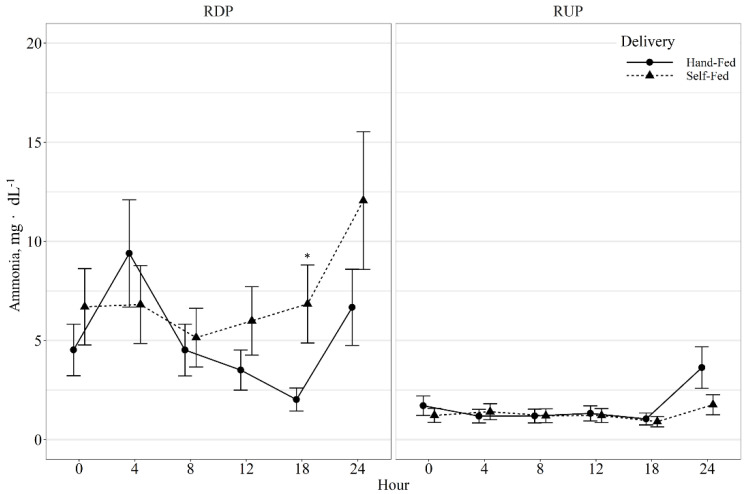
Ruminal ammonia concentrations of beef cows fed hand-fed or self-fed rumen degradable (RDP) or rumen undegradable (RUP) protein supplements with low-quality forage at 08:00. Ruminal ammonia concentrations were influenced by delivery method × protein type × hour post-feeding interaction (*p* < 0.01), and differences within hour post-feeding are denoted by *, timepoints without a common letter are different (*p* < 0.05).

There were no protein type or delivery method effects on acetate, propionate, butyrate, isobutyate, isovalerate, acetate:propionate ratio, or total VFAs (*p* ≥ 0.19; Table 5). Ruminal ammonia displayed a protein type × delivery × hour interaction (*p* < 0.01; Figure 1), with RDP-SF having greater (*p* < 0.01) ammonia concentrations at 18-h post-feeding than RDP-HF, with a tendency (*p* = 0.09) for RUP-HF to have greater ammonia concentrations than RUP-SF at 24-h post-feeding. However, the RDP cows had greater (*p* ≤ 0.04) ammonia concentrations than RUP, regardless of delivery method, at each hour. There was a delivery × hour interaction for valerate (*p* = 0.04); however, there was no difference (*p* ≥ 0.12) between delivery methods within each hour. There tended to be a delivery × protein type interaction (*p* = 0.10) for isovalerate, where RDP-SF tended to have greater isovalerate concentrations than RDP-HF, with no effect of delivery method for RUP.

**Figure 2:**
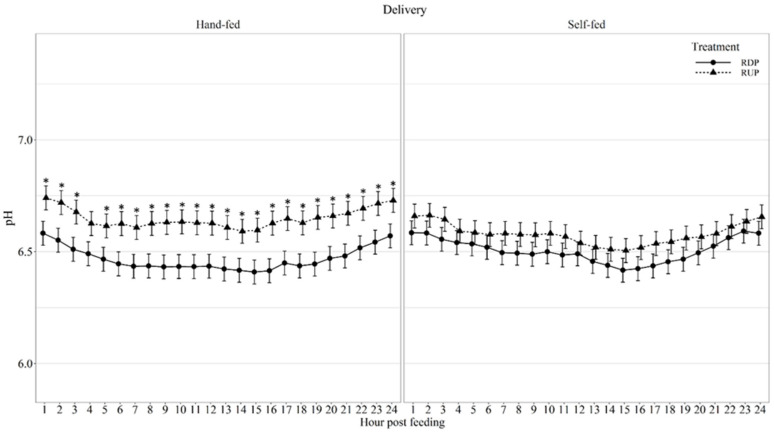
Diurnal ruminal pH patterns of beef cows fed hand-fed or self-fed rumen degradable (RDP) or rumen undegradable (RUP) protein supplements with low-quality forage at 08:00. Ruminal pH was influenced by delivery method × protein type × hour post-feeding interaction (*p* < 0.01), and differences (*p* ≤ 0.05) within hour post-feeding are denoted by *.

**Figure 3:**
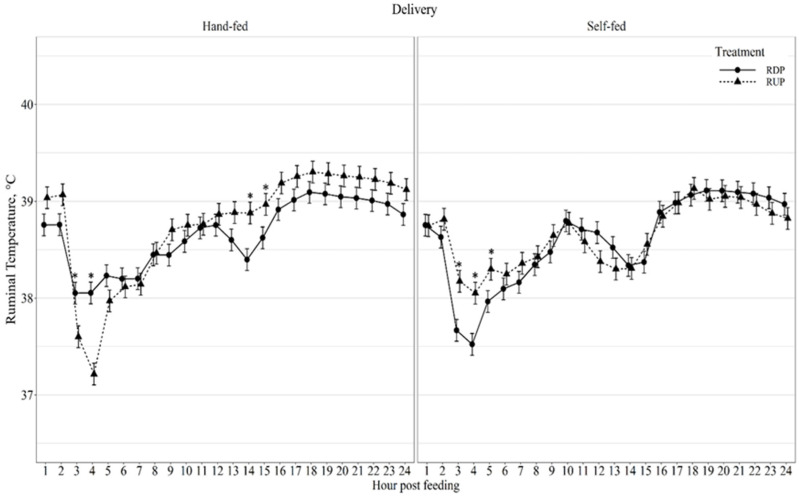
Diurnal ruminal temperature (°C) patterns of beef cows fed hand-fed or self-fed rumen degradable (RDP) or rumen undegradable (RUP) protein supplements with low-quality forage at 08:00. Ruminal temperature was influenced by delivery method × protein type × hour post-feeding interaction (*p* < 0.01), and differences (*p* ≤ 0.05) within hour post-feeding are denoted by *.

**Figure 4:**
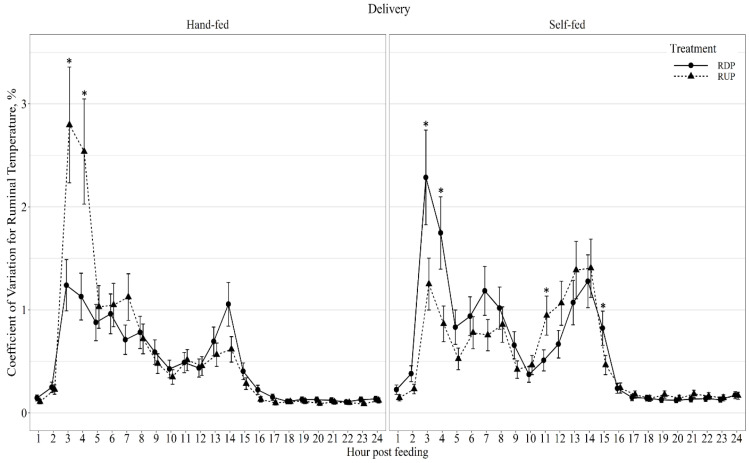
Diurnal ruminal temperature coefficient of variation (CV, %) patterns of beef cows fed hand-fed or self-fed rumen degradable (RDP) or rumen undegradable (RUP) protein supplements with low-quality forage at 08:00. Ruminal temperature CV was influenced by delivery method × protein type × hour post-feeding interaction (*p* < 0.01), and differences (*p* ≤ 0.05) within hour post-feeding are denoted by *.

Diurnal ruminal pH displayed a delivery method × protein type × hour interaction (*p* < 0.01; Figure 2), in which RDP-HF produced lower pH than RUP-HF at all hours, except hour 4 post-feeding. Diurnal pH coefficient of variation (CV) displayed a protein type effect (*p* = 0.02; Table 5) as RDP-supplemented cows had greater variation in pH than RUP-supplemented cows. Diurnal ruminal temperature displayed a delivery method × protein type × hour interaction (*p* < 0.01; Figure 3). Ruminal temperature was greater (*p* ≤ 0.03) in RDP-HF cows than RUP-HF cows at 3-, 4-, 14-, and 15-h post-feeding. Ruminal temperature was greater (*p* ≤ 0.04) in RUP-SF cows at 3-, 4-, and 5-h post-feeding than RDP-SF cows. Diurnal ruminal temperature coefficient of variation (CV) displayed a delivery method × protein type × hour interaction (*p* < 0.01; Figure 4). Ruminal temperature CV was greater (*p* ≤ 0.01) in RUP-HF cows compared to RDP-HF cows at 3- and 4-h post-feeding. Ruminal temperature CV was greater (*p* ≤ 0.05) in RDP-SF cows at 3-, 4-, and 15-h post-feeding and reduced (*p* = 0.03) at 11-h post-feeding, compared with RUP-SF cows.

## 4. Discussion

Supplement intake was greatest for SF animals, as HF animals were given an allotted amount of supplement; therefore, tending to increase their total dry matter intake. Similar to previous research, SF cattle had variation in supplement intake [25,26,27], which led to increased total feed intake in the current study. The tendency for RDP-SF to increase DM digestibility may be attributed to RDP being a protein source for microbes, promoting their growth and efficiency [4] and, therefore, increasing digestibility. When lambs were fed low-quality forage and supplemented to meet RDP requirements, or to meet 50, 100, or 150% of RUP requirements, NDF intakes were not impacted by protein type, but tended to increase with increasing amounts of RUP [28]. Similarly, RUP-SF animals had increased NDF intakes; however, so did RDP-SF animals, which is indicative of the greater SF intakes. Nellore heifers supplemented with RUP at 25 or 40% of CP had no impacts on DMI, or DM and NDF digestibility [9]. Similarly, in the current study, DM digestibility was not impacted by treatment; however, NDF digestibility was greater in RDP-SF cows than in RDP-HF cows. Periparturient cows supplemented with low, medium, and high levels of RUP compared to a control, non-supplemented, group had greater NDF digestibility compared to the control group [10]. As the supplements had the same amount of RDP, this suggests that control cows may have been RDP deficient. These results suggest that RDP, provided in self-fed form, may enhance the fiber digestion of low-quality forages. 

Beef cows consuming low-quality forages had increased water intake when fed increasing amounts of salt [29]. Likewise, in the current study, RDP-SF animals had greater water intakes. Animals consuming SF supplements had greater supplement intakes, and because the supplement was salt-limited, they consumed more salt. The RDP-SF cows had the greatest supplement intake, which is likely why those cows had increased water intakes. However, animals with increased salt intakes, and that had increased water intakes, have also been reported to have increased rumen liquid fill [29,30]. In contrast, in the current study the animals that had increased water intake did not have increased rumen liquid volume, likely because there were no differences in fluid flow rates between treatments. 

In steers supplemented with RDP, ammonia concentrations increased with increasing amount of RDP [4]. Similarly, in the current study, RDP-SF cows had the greatest ruminal ammonia concentrations. Animals consuming high levels of salt-limited protein supplements have been reported to have decreased ruminal ammonia and VFA concentrations [31], likely due to an increase in salt intake. In the current study, SF animals had increased supplement intakes and because the supplement was salt-limited, they had increased salt intakes. However, SF animals did not exhibit decreases in ruminal ammonia or VFA concentrations.

Both cows and steers supplemented with increasing amounts of RDP had increasing amounts of valerate, isovalerate, and isobutryrate [3,4]. Likewise, in the current study, RDP supplemented animals had greater levels of valerate and tended to have greater levels of isovalerate, compared to RUP-supplemented animals. In contrast, there were no differences in isobutyrate or total VFA concentrations, as Köster et al. [3] reported. Wickersham et al. [4] reported that steers also had increased propionate amounts as RDP amount increased, and a decrease in acetate. There were no effects of protein type on propionate or acetate amount in the current study. Propionate is the major gluconeogenic precursor (60–75%), while valerate and isobutyrate collectively contribute 5–6% [32]. As we observed an increase in valerate in RDP-supplemented animals, this may have led to an increase in gluconeogenesis; however, since there was no difference in propionate levels, it is highly unlikely gluconeogenesis increased. 

When comparing the impacts of RDP and RUP supplementation on rumen characteristics, Atkinson et al. [28] reported no impacts of protein degradability on rumen pH. However, when steers were supplemented with low amounts of RUP, they had a decrease in pH when compared to steers supplemented with medium or high levels of RUP. In the current study, there was a delivery method by protein type by hour interaction, where RUP-HF cows had higher pH than RDP-HF cows at all time points, except 4-h post-feeding. This may be due to the RDP group having produced greater levels of ruminal ammonia at all time points, as ammonia indicates free hydrogen ions and lower pH [33]. There was also a delivery type by hour interaction, where SF animals had lower pH values compared to HF animals at 23-h, likely due to the availability of the SF supplements, to be consumed throughout the day, unlike the HF supplements. 

Little research has been conducted on the impacts of protein type or delivery method on diurnal ruminal temperature or diurnal ruminal temperature CV. Similar to the current results, ruminal temperature decreased 3- to 4-h post-feeding in dairy cows fed a forage-based diet [34]. In the current study, ruminal temperature also declined between 14- and 15-h post-feeding, which was not observed by Kimura et al. [34]. Self-fed cows may have contributed to the variation in temperature throughout the 24-h period, due to the constant availability of the protein supplement. The largest variation in ruminal temperature occurred 3- to 4-h post-feeding, which was similar to ruminal pH. Ruminal temperature peaks 18-h post-feeding and remains relatively constant until feeding gain, which is also indicated by the reduced variation in ruminal temperature from 17- to 24-h.

## 5. Conclusions

Self-fed supplements tend to increase dry matter intake, due to increases in supplement intake. Self-fed supplements also increase fiber intake, valerate, and tend to increase isovalerate. Valerate is a gluconeogenic precursor [32], and this increase in concentrations has the potential to increase gluconeogenic rates. Self-fed rumen degradable protein supplements increase the efficiency of utilization of low-quality forages. The results from this research provide additional information on how rumen degradable protein and rumen undegradable protein can impact nutrient digestion in beef cows consuming low-quality forage. 

## Figures and Tables

**Table 1:** Supplement nutrient analysis of supplements offered to fistulated 2- and 3-year-old beef cows.

Item Analyzed	RDP-HF ^1^	RDP-SF ^1^	RUP-HF ^1^	RUP-SF ^1^
Dry matter, %	87.7	86.6	77.4	77.3
Analyzed nutrient composition, % DM basis				
Crude protein	37.7	37.0	27.6	27.8
ADICP ^2^	2.4	1.2	1.7	2.5
Soluble protein, % CP	27	15	16	13
Degradable protein, % CP	54	63	35	30
Acid detergent fiber	18.7	5.5	8.1	6.4
Neutral detergent fiber	30.1	8.8	11.7	8.8
Lignin	7.6	1.1	3.4	2.6
Non-fiber carbohydrates	21.4	22.1	16.4	16.6
Starch	3.0	0.9	3.0	2.5
Crude fat	3.64	1.17	7.42	7.20
Ash	14.91	28.0	35.0	35.3
Total digestible nutrients	68.0	60.0	57.0	57.0
Macrominerals composition, % DM basis				
Calcium	2.68	2.77	2.07	1.98
Phosphorus	1.52	1.91	2.25	2.04
Magnesium	0.62	0.80	3.10	2.96
Potassium	1.34	2.5	2.10	1.84
Sodium	0.26	4.30	5.84	6.15
Sulfur	0.85	0.73	1.49	1.23
Microminerals composition, mg kg^−1^				
Iron	485	1060	1000	852
Zinc	680	1300	1460	1530
Copper	216	222	788	848
Manganese	303	726	1000	1060
Molybdenum	1.3	8.1	2	1.5

^1^ Protein type of rumen degradable protein (RDP) or rumen undegradable protein (RUP), and delivery method of hand-fed (HF) or self-fed (SF). ^2^ Acid detergent insoluble crude protein.

**Table 2:** Nutrient analysis of hay offered to fistulated 2- and 3-year-old beef cows.

Item Analyzed	
Dry matter, %	85.16
Analyzed nutrient composition, % DM basis	
Crude protein	7.21
Total digestible nutrients	57.58
Acid detergent insoluble crude protein	1.07
Soluble protein	39.94
Acid detergent fiber	31.23
Neutral detergent fiber	61.01
Lignin	7.13
Fat	1.99
Ash	7.22
NDFD ^1^, % NDF	40.8
uNDF240h ^2^, % NDF	38.1
Macromineral composition, % DM basis	
Calcium	0.26
Phosphorus	0.14
Magnesium	0.16
Potassium	1.62
Sodium	0.03
Chloride	0.44
Sulfur	0.12

^1^ Neutral detergent fiber digestibility. ^2^ Undigestible neutral detergent fiber based on a 240 h in vitro.

**Table 3:** Impacts of supplement delivery method and protein type on intake and fiber digestion of 2- and 3-year-old beef cows fed low-quality forage.

Item	RDP ^1^		RUP ^1^		SEM ^3^	*p*-Value ^4^		
	HF ^2^	SF ^2^	HF	SF		D	P	D × P
Dry matter intake, kg d^−1^	11.61	13.25	12.11	12.91	0.59	0.07	0.56	0.49
Dry matter intake, g kg BW^−1^ d^−1^	21.53	24.75	22.54	24.54	1.82	0.24	0.70	0.74
Supplement intake, kg d^−1^	0.83	2.34	0.76	1.40	0.36	0.01	0.89	0.24
Supplement intake, g kg BW^−1^ d^−1^	1.54	3.91	1.41	2.20	0.58	0.12	0.03	0.82
Forage intake, kg d^−1^	10.78	10.91	11.35	11.52	0.60	0.89	0.52	0.97
Forage intake, g kg BW^−1^ d^−1^	19.98	20.24	21.11	21.72	1.27	0.89	0.54	0.89
NDF intake, kg d^−1^	6.63	7.81	6.89	7.32	0.37	0.04	0.63	0.33
Dry matter digestibility, %	49.10	51.17	51.37	48.05	1.44	0.32	0.28	0.08
NDF digestibility, %	42.03 ^a^	50.27 ^b^	46.86 ^ab^	43.11 ^ab^	2.61	0.05	0.20	0.04
Water intake, L d^−1^	48.38 ^a^	72.60 ^b^	52.61 ^ab^	52.81 ^ab^	4.87	<0.01	0.55	0.03
uNDF Fill, kg	4.47	5.11	4.89	4.12	0.47	0.35	0.54	0.15
uNDF Fill, g kg BW^−1^	8.22	9.29	9.28	7.7	0.90	0.38	0.42	0.15
uNDF Retention, h	62.98	73.7	68.34	56.52	6.82	0.29	0.59	0.12
uNDF Passage, % h^−1^	1.64	1.42	1.49	1.84	0.17	0.38	0.55	0.12

^1^ Protein type of rumen degradable protein (RDP) or rumen undegradable protein (RUP). ^2^ Delivery method of hand-fed (HF) or self-fed (SF). ^3^ Pooled standard error of the means presented. ^4^ Delivery (D): delivery method of HF vs. SF; protein (P): type of protein fed (RDP vs. RUP); and the interaction of delivery method and protein type (D × P). ^a,b^ Means that lack common superscripts differ for delivery × protein (*p* < 0.05).

**Table 4:** Impacts of supplementation on rumen kinetics of 2- and 3-year-old beef cows fed low-quality forage.

Item	RDP ^1^		RUP ^1^		SEM ^3^	*p*-Value ^4^		
	HF ^2^	SF ^2^	HF	SF		D	P	D × P
Fluid flow rate, L h^−1^	1.89	2.72	2.75	2.92	0.23	0.03	0.02	0.18
DM volume, L	12.09	13.53	13.69	12.23	1.19	0.41	0.36	0.25
Fluid volume, L	81.06	84.77	99.14	90.92	6.31	0.69	0.07	0.36

^1^ Protein type of rumen degradable protein (RDP) or rumen undegradable protein (RUP). ^2^ Delivery method of hand-fed (HF) or self-fed (SF). ^3^ Pooled standard error of the means presented. ^4^ Delivery (D): delivery method of HF vs. SF; Protein (P): type of protein fed (RDP vs. RUP); and the interaction of delivery method and protein type (D × P).

**Table 5:** Impacts of supplement deliver method, protein type, and hour on ruminal pH and VFA concentrations in 2- and 3-year-old beef cows fed low-quality forage.

Item	RDP ^1^		RUP ^1^		SEM ^3^	*p*-Value ^4^						
	HF ^2^	SF ^2^	HF	SF		D	P	H	D × P	D × H	P × H	D × P × H
Average daily ruminal pH	6.47	6.51	6.65	6.58	0.053	0.62	0.02	<0.01	0.31	<0.01	<0.01	<0.01
Average daily ruminal pH CV, %	1.14	1.31	0.90	1.03	0.085	0.15	0.04	0.08	0.84	0.11	0.25	0.40
Average daily ruminal temp., °C	38.66	38.60	38.77	38.64	0.095	0.65	0.41	<0.01	0.72	<0.01	<0.01	<0.01
Average daily ruminal temp. CV, %	2.09	2.45	2.41	2.04	0.201	0.20	0.26	<0.01	0.07	0.72	0.01	<0.01
Ammonia, mg dL^−1^	4.57	6.99	1.52	1.26	0.89	0.33	0.02	<0.01	0.21	<0.01	<0.01	<0.01
Volatile fatty acids, mol 100 mol^−1^												
Acetate	66.06	65.73	67.40	66.13	0.68	0.87	0.22	<0.01	0.85	0.57	0.93	0.41
Propionate	18.14	17.94	17.51	18.47	0.44	0.47	0.44	<0.01	0.32	0.96	0.35	0.40
Isobutyrate	1.40	1.67	1.29	1.30	0.10	0.21	0.40	<0.01	0.18	0.12	0.80	0.17
Butyrate	9.86	9.51	10.10	10.47	0.29	0.19	0.90	<0.01	0.32	0.63	0.59	0.71
Isovalerate	1.69	2.38	1.40	1.27	0.19	0.18	0.31	<0.01	0.10	0.30	0.39	0.13
Valerate	2.03	2.12	1.84	2.02	0.08	0.53	0.04	<0.01	0.72	0.04	0.73	0.17
Acetate:propionate	3.59	3.67	3.87	3.59	0.12	0.57	0.35	<0.01	0.44	0.95	0.55	0.45
Total VFA, mM	89.51	91.21	89.67	88.77	3.03	0.21	0.75	<0.01	0.67	0.74	0.99	0.90

^1^ Protein type of rumen degradable protein (RDP) or rumen undegradable protein (RUP). ^2^ Delivery method of hand-fed (HF) or self-fed (SF). ^3^ Pooled standard error of the means presented. ^4^ Delivery (D): delivery method of HF vs. SF; protein (P): type of protein fed (RDP vs. RUP); and possible interactions of hour (H), delivery method and protein type.

## Data Availability

The data are available in Tables S1–S3.

## Supplementary Materials

The following are available online at https://www.mdpi.com/article/10.3390/ani11113011/s1, Supplementary materials include the raw data that can be found in Table S1: Temperature and pH data from a rumen indwelling bolus for delivery method and protein type, Table S2: Dry matter intake, digestibility, rumen fill, fluid flow rate, and undigestible neutral detergent fiber data based on delivery method and protein type, and Table S3: Ammonia and volatile fatty acid data for cattle based on delivery method and protein type.
